# Transcriptomic Profiling of Gametogenesis in Triploid Pacific Oysters *Crassostrea gigas*: Towards an Understanding of Partial Sterility Associated with Triploidy

**DOI:** 10.1371/journal.pone.0112094

**Published:** 2014-11-06

**Authors:** Nolwenn M. Dheilly, Aude Jouaux, Pierre Boudry, Pascal Favrel, Christophe Lelong

**Affiliations:** 1 UNICAEN, UMR BOREA “Biologie des ORganismes et Ecosystèmes Aquatiques”, MNHN, UPMC, CNRS-7208, IRD-207, Caen, France; 2 Ifremer, UMR 6539 LEMAR “Laboratoire des sciences de l’Environnement MARin”, UBO/CNRS/IRD/Ifremer, Plouzané, France; University of Innsbruck, Austria

## Abstract

**Background:**

Triploidy can occur in many animal species but is often lethal. Among invertebrates, amphibians and fishes, triploids are viable although often sterile or infertile. Most triploids of the Pacific oyster *Crassostrea gigas* are almost sterile (named “3nβ”) yet a low but significant proportion show an advanced gametogenesis (named “3nα”). These oysters thus constitute an interesting model to study the effect of triploidy on germ cell development. We used microarrays to compare the gonad transcriptomes of diploid 2n and the abovementioned triploid 3nβ and 3nα male and female oysters throughout gametogenesis.

**Results:**

All triploids displayed an upregulation of genes related to DNA repair and apoptosis and a downregulation of genes associated with cell division. The comparison of 3nα and 3nβ transcriptomes with 2n revealed the likely involvement of a cell cycle checkpoint during mitosis in the successful but delayed development of gonads in 3nα individuals. In contrast, a disruption of sex differentiation mechanisms may explain the sterility of 3nβ individuals with 3nβ females expressing male-specific genes and 3nβ males expressing female-specific genes.

**Conclusions:**

The disruption of sex differentiation and mitosis may be responsible for the impaired gametogenesis of triploid Pacific oysters. The function of the numerous candidate genes identified in our study should now be studied in detail in order to elucidate their role in sex determination, mitosis/meiosis control, pachytene cell cycle checkpoint, and the control of DNA repair/apoptosis.

## Background

Polyploidy occurs when mitotic or meiotic accidents induce the formation of gametes with more than one set of chromosomes. Triploidy results from the fusion of haploid and diploid gametes and can occur among all animal species. In mammals and birds, the odd number of chromosomes severely impacting mitosis and meiosis, frequently leads to lethal abnormalities [1], [2], [3], making triploids less common in the wild. In invertebrates, amphibians and fishes, triploids are often difficult to distinguish from diploids, however, in some species, they have a larger body size and faster growth than diploids. This is often however at the expense of gonadal development [4], [5], [6], [7] since in most species, triploids are either sterile or infertile. Differences in growth and reproductive capacity between diploid and triploids have often been used in aquaculture to increase production or to reduce genetic impact of escapes from cultured stock [8], [9], [10].

Since its first induction in the early 1980’s [11], triploidy has become an important innovation in the oyster aquaculture industry. Triploid oysters can be produced by two methods: by inhibiting polar body formation after fertilization or by mating diploid and tetraploid broodstock. Recently, in the Pacific oyster *Crassostrea gigas* (Thunberg, 1793) and the American oyster *Crassostrea virginica* (Gmelin, 1791), triploid oysters have been produced and grown in large quantities using the second method [12], [13]. They present an important aquacultural benefit mainly by virtue of their faster growth [14], [15], their strong reduction of gonad development [16], [17], [18] and their better survival [19]. The Pacific oyster is an alternating and irregular protandrous hermaphrodite, meaning that most (≈20% [19]) individuals mature first as males and then change sex several times. While sex ratio is not affected by triploidy in *C. gigas* (resulting either from chemical induction or from crosses between tetraploid and diploid parents), the number of simultaneous hermaphrodites and undifferentiated individuals is significantly higher [18].

Most triploid oysters display highly reduced gonadal development compared with diploid oysters [18], and thus are commonly referred to as being sterile and are considered as genetically confined. However, 25% of triploid oysters have been shown to produce a significant number of gametes [17], [20], [21] and revert to diploid-triploid mosaics [22]. Such non-sterile triploid oysters have been named triploid alpha (3nα) oysters, differentiating them from those producing only a few gametes called triploid beta (3nβ) [20]. Interestingly, gametes of triploid oysters can reach functional maturity [23] and when fertilized, can develop into some viable progeny, though the proportion of aneuploids is high [23], [24].

The vast majority of studies on triploid gametogenesis have relied solely on qualitative or quantitative histological observations. Data able to unravel the mechanisms behind the physiological differences in gametogenesis of triploid individuals is lacking. Because both sterile and partially fertile triploid oysters exist, oysters constitute a unique model to study the effect of triploidy on germ cell development. The current study was designed in order to provide more information on the molecular mechanisms governing the differences in fertility between alpha and beta triploid Pacific oysters *Crassostrea gigas*. We employed the custom microarray described and validated in Dheilly et al. [25] and compared the gonadal transcriptomes with the previously published transcriptome of diploid oysters [26].

## Methods

### Production of biological material and rearing conditions

Triploid juveniles were obtained from the commercial hatchery France Naissain (Vendée, France) and then maintained at Blainville sur mer (Manche, France). They had been bred by crossing tetraploid males and diploid females [12] and ploidy was checked by flow cytometry (Epics XL Beckman Coulter) [18]. Triploid oysters were sampled 3 times between September 2009 and July 2010. Diploid oysters were sampled 8 times between November 2008 and September 2009 in Locmariaquer (Brittany, France) in marine coastal areas specifically dedicated for marine culture. On each sampling, their gonads were immediately dissected and frozen in liquid nitrogen until later use. Then, gonad tissue was sampled for each individual and either homogenized in Tri-reagent (Sigma) (100 mg/mL) and stored at −80°C for total RNA extraction, or fixed for histological analysis. Gonadal development stages and sex were strictly selected by histological methods according to the 5 previously described stages [27], [28], with particular attention made to eliminate ambiguous cases such as hermaphrodites.

### RNA extraction

Four individual gonad samples for each gonad developmental stage and sex of diploid oysters were prepared for microarray analysis, except for stage 0 for which RNA from 8 individual gonads were sampled [26]. Samples were named as follows: 2n stage 0 (2n_St0, n = 8), 2n stage I male (2n_StI_M, n = 4), 2n stage I female (2n_StI_F, n = 4), 2n stage II male (2n_StII_M, n = 4), 2n stage II female (2n_StII_F, n = 4), 2n stage III male (2n_StIII_M, n = 4) 2n stage III female (2n_StIII_F, n = 4) [26]. For the triploids, processed gonads included four undifferentiated stage 0 (3n St0), 8 alpha (3n StIα) and 8 beta (3n StIβ) stage I, and finally 4 of each sex for both alpha (3n F StIIIα and 3n M StIIIα) and beta (3n F StIIIβ and 3n M StIIIβ) stage III. Samples of gonad tissue in Tri-reagent (Sigma) were solubilized using a needle (0.9 mm). Total RNA was then isolated with a Nucleospin RNAII column (Macherey Nagel), following manufacturer instructions. The presence of residual genomic DNA was controlled by performing PCR on the *actin* gene from RNA samples before RT (minus RT control). The presence of an amplification product in the “-RT” control was indicative of contaminating genomic DNA in the sample. Any residual DNA was removed by a supplementary cleaning with Nucleospin RNA clean up (Macherey Nagel) isolation column. RNA integrity was checked on the Agilent bioanalyzer using RNA nanochips and Agilent RNA 6000 nanoreagents (Agilent Technologies, Waldbronn, Germany) according to manufacturer instructions without consideration for the RNA integrity number (RIN) [25]. RNA concentrations were measured at 260 nm using an ND-1000 spectrophotometer (Nanodrop Technologies) using the conversion factor 1 OD = 40 µg/mL ssRNA. Samples were stored at −80°C until use. One sample, 3n M_StIIIβ had poor RNA quality and was not processed further.

### cDNA microarray

RNA amplification, labeling and one color hybridization were performed as previously described [25] using the custom and validated design [25], [26] and with the Low Input Quick Amp labeling kit (Agilent), Qiagen’s RNeasy mini spin columns and the Agilent Gene expression hybridization kit. We employed a custom oligonucleotide microarray containing 31,918 ESTs described and validated in Dheilly et al. [25]. Slides were scanned on an Agilent Technologies G2565AA Microarray Scanner system at 5 µm resolution. Thirty-two separate arrays were used to study the four gametogenetic stages of male and female diploid oysters [26]. This dataset has previously been made available through NCBI via the Gene Expression Omnibus (GEO) data repository (GEO accession GSE27955; (http://www.ncbi.nlm.nih.gov/geo/) [26]. A total of 35 new separate arrays were used and the complete dataset (raw data and normalized values, including diploid samples) was made available through NCBI via the Gene Expression Omnibus (GEO) data repository (GEO accession GSE40855). Feature extraction and data normalization were conducted with Agilent Feature Extraction software 6.1 using the default/recommended normalization methods as previously described [25], [26].

### Correction and normalization methods

Feature extraction and data normalization were conducted with Agilent Feature Extraction software 6.1 using the default/recommended normalization methods described in previous studies employing the same *C. gigas* arrays [25], [26]. Raw data extraction and normalization were conducted with Agilent Feature Extraction software 6.1 using the default/recommended normalization methods. A matrix of gene expression levels was generated in which each row corresponded to a different gene and each column to one oyster gonad sample. The expression level of each gene was then logarithmically transformed and centered (relative to zero) to enable the use of relative variations rather than absolute values for interpretation, as previously described [25].

### Statistical analysis

A principal component analysis (PCA) was performed using GeneANOVA software with default/standard parameters [29]. The proportion of variance for each principal component and the cumulative variance were obtained. The four components with the highest proportion of variance were used to draw 2D score plots (XLStat; Addinsoft). Student’s t-test and a one-way ANOVA parametric test were then used to identify the genes differentially expressed between 2n and 3n, with p-values<0.01 and Bonferroni adjustment using TMeV 4.6.0 software [30], [31].

### Bioinformatics analysis

The functional annotation of differentially expressed genes was performed using Blast 2 GO as follows: i) an initial annotation with BLASTX (against the non-redundant NCBI database; e-value at 1.10^−6^); ii) assignment of Gene Ontology terms (GO; http://www.geneontology.org/); iii) protein domain searches using InterProscan; and iv) enzyme annotation using the Kyoto Encyclopedia of Genes and Genomes (KEGG). GO term enrichment was performed on the list of genes differentially expressed between diploid and triploids according to a Fisher exact test (p<0.05).

Data were further interpreted using Ingenuity Pathways Analysis (IPA) (Ingenuity Systems, Redwood City, CA, USA) (http://www.ingenuity.com). The list of significantly regulated genes, as selected by the statistical analysis described above, was loaded into IPA to generate networks of genes associated with particular biological functions and molecular processes.

### Real time quantitative PCR

Two hundred and fifty nanograms of total RNA from each sample were reverse transcribed using 200 U of MMuLV-RT (Moloney Murine Leukemia Virus Reverse transcriptase, Promega) in the presence of 20 U of RNase inhibitor (RNasin, Promega), 0.5 mM of RNAse free dNTP and in the appropriate buffer (Promega). Selected genes are listed in File S1 with annotations and primer sequences. The high stability of the reference gene used in this study, EF1α (*Crassostrea gigas* EFα1 AB122066), was previously identified by Dheilly et al. [25] and was further confirmed in the 2n and 3n oyster gonad samples used in the present study.

## Results

### Ploidy, sex and gametogenesis: principal component analysis

**Figure 1:**
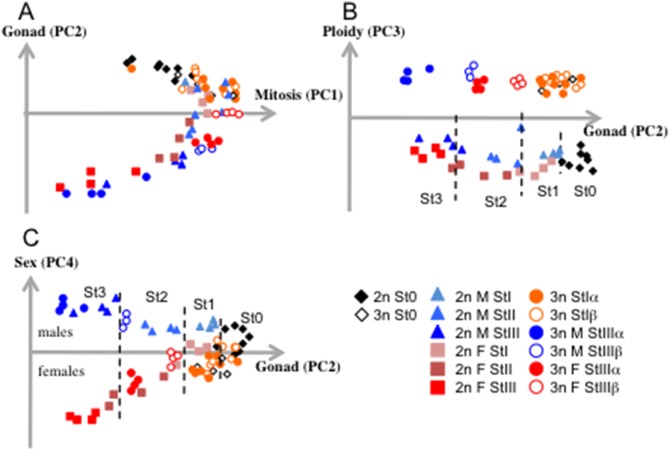
2D score plots (A, B and C) obtained by the principal components analysis using GeneANOVA with default/standard parameters and XLStat (Addinsoft) of all 31,918 transcripts in the 32 diploid oyster gonads and the 35 triploid oyster gonads (A: Mitosis (PC1) vs Gonad stages (PC2); B: Gonad stages (PC2) vs Ploidy (PC3); C: Gonad stages (PC2) vs Sex (PC4); PC: principal component).

We performed a microarray analysis on a total of 67 individual diploid and triploid gonads grouped as follows: 2n stage 0 (2n_St0, n = 8), 2n stage I male (2n_StI_M, n = 4), 2n stage I female (2n_StI_F, n = 4), 2n stage II male (2n_StII_M, n = 4), 2n stage II female (2n_StII_F, n = 4), 2n stage III male (2n_StIII_M, n = 4), 2n stage III female (2n_StIII_F, n = 4), 3n stage 0 (3n St0, n = 4), 3n stage Iα (3n StIα, n = 8), 3n stage Iβ (3n StIβ, n = 8), 3n male stage IIIα (3n M StIIIα, n = 4), 3n male stage III β (3n M StIIIβ, n = 3), 3n female stage IIIα (3n F StIIIα, n = 4), and 3n female stage IIIβ (3n F StIIIβ, n = 4) (for β individuals, we selected only complete blocked animals, described as the β1 subcategory in [20] and homogeneous females and males at stage III, i.e. with approximately the same reproductive effort). The comparative analysis of gametogenesis in male and female diploid oysters has previously been analyzed and published by Dheilly et al. [26] In the present study, all samples were normalized together before analysis, as previously described [25], [26]. Differences among individual gonad transcriptomes were analyzed with respect to four main factors: sex (male or female), gonad developmental stage (St0 to StIII), ploidy (2n or 3n) and subgroup of triploids (3nα or 3nβ). Score plots, using the four principal components of our PCA analysis, are shown in Figure 1. Principal component 1 (PC1) showed a very high proportion of variance (89.8%) and discriminated according to stage of mitosis. Diploids and triploids had the highest PC1 values at Stage I and the lowest at stage III. Principal components 2, 3 and 4 discriminated individuals according to their gonad developmental stage (from Stage 0 to III; 1.43%), ploidy (3n versus 2n; 0.94%) and sex (males versus females; 0.70%) respectively. To confirm the validity of the expression profiles obtained in triploid individuals, we further tested the expression of 8 genes on 2n and 3n individuals by RT-qPCR (File S1). Again, we observed a very high congruence of the expression profiles, with the exception of the variance of RT-qPCR data which was higher in lowly expressed genes.

Analysis of 2D score plots (Figure 1B) showed two distinct clouds of samples representing 2n and 3n individuals (PC3) and revealed that differences between 2n and 3n gonads were conserved from St0 to StIII and were similar in males and females. Principal component 4 discriminated two subgroups corresponding to male and female among both 2n and 3n (Figure 1D). However, divergence in expression patterns between male and female gonads appeared to increase during gonad maturation, with F and M StIII showing the most distinct expression profiles (PC2, Figure 1C). Indeed, differences between 3nα and 3nβ could not be observed in StI but were significant in StIII individuals (PC2, Figures 1B and C). We found a discordance between F and M triploids at StIII, with 3n M StIIIα transcriptomes closely resembling 2n M StIII transcriptomes while 3n M StIIIβ clustered closely to 2n M StII (Figures 1B and C), and with 3n F StIIIα transcriptome resembling 2n F StII samples while 3n F StIIIβ clustered more closely to 2n F StI (Figure 1C).

### Diploid *versus* triploid

In order to identify genes that are differentially regulated in 3n oyster gonads, we performed a Student’s t-test (with Bonferroni adjustment) on all 2n versus all 3n gonads, disregarding the sex or developmental stage. This comparison identified 1,911 genes that were differentially expressed between diploids and triploids, with a threshold of adj p<0.01 (File S2). In total, 505 genes and 1,406 genes were upregulated and downregulated in 3n, respectively. Very few of the differentially expressed genes have previously been found to be involved in gametogenesis in 2n (File S2) [26]. While we did extract Gene Ontology terms, Fisher exact test (p<0.05) was unsuccessful at identifying differentially represented Gene Ontology terms. However, a functional classification performed with Ingenuity Pathway Analysis (IPA) revealed a significant differential representation of some metabolic and signaling pathways within the lists of upregulated and downregulated genes (File S3).

Following ingenuity pathway analysis, upregulated genes in 3n shared networks with the NFκB complex and RNA polymerase II. Other upregulated genes included *estrogen receptor 1*, encoding a nuclear factor involved in vertebrate reproductive system control, *caspase 7* involved in apoptosis execution, and *hnf4a* (hepatocyte nuclear factor 4), encoding a steroid hormone receptor involved in the regulation of lipid metabolism. Among the most upregulated genes, we found the *ap2 complex*, *profillin*, *caspase 3*, *histidine triad nucleotide-binding protein 1* (*hint1*), *anamorsin*, *jagged* and *notch* genes.

Downregulated genes in triploids encoded proteins with links to *rock*, encoding an actin cytoskeleton-signaling molecule. Further analysis of this network revealed a downregulation of *protein phosphatase 1* (*pp1*), *myosin light chain kinase* (*mylk*), *LIM domain kinase* (*limk*), *paxillin* (*pxn*), *talin*, *integrins*, *myosin*, *wave*, *focal adhesion kinase* (*fak*), *phosphatidyl-inositol 3 kinase* (*pi3k*), and *laminin*, together involved in actin and actomyosin assembly necessary for cell division and proliferation. On the edge of the network, a second group of genes appeared connected to *smarca4*, involved in chromatin remodeling and transcriptional regulation, and *rna polymerase II*. Among the most downregulated genes, we found the gene *peroxisomal targeting signal import receptor* (*pxr1*).

### Expression of sex-specific genes

**Figure 2:**
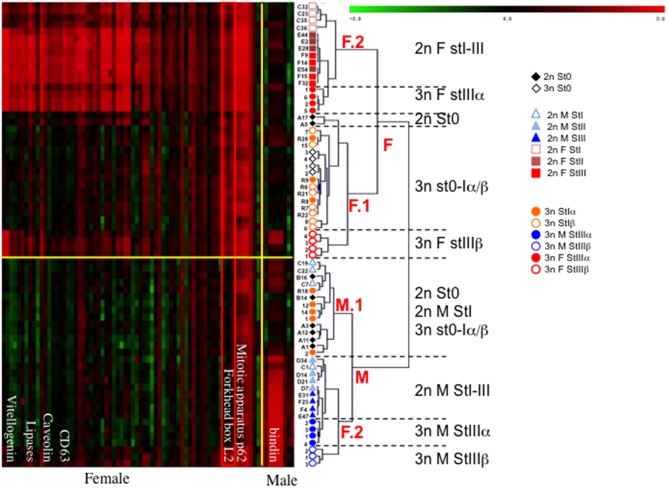
Hierarchical clustering using Pearson’s correlation was performed using TMeV 4.6.0 software on the statistically significant transcripts (one-way ANOVA parametric test with a p-value cut-off of 0.01 and Bonferroni adjustment). Complete linkage reveals the positioning of triploid oyster gonads among diploid oyster gonads. Genes employed were identified as sex specific in Dheilly et al. [26].

Using a list of sex-specific genes previously determined by comparing the transcriptomes of male and female diploid oysters [26], we performed a hierarchical clustering using Pearson’s correlation and complete linkage and observed the positioning of 3n transcriptomes among those of 2n (Figure 2). Two main branches separated the groups of individuals with distinct gene expression profiles, expressing high and low levels of male (M) and female (F) specific genes. Within each branch, we found two sub-branches that separated the M branch into early male gametogenetic stage 1 and some of 2n stage 0 (M.1) and late male gametogenetic stages 2 and 3 (M.2), and the F branch into some 2n stage 0 (F.1) and female gametogenetic stages 1 to 3 (F.2). Triploid stage 0 grouped exclusively into M.1, while the stages 1α and β divided into F.1 and M.1. In M.2, 3n M StIIIα clustered closely to 2n M StII and StIII, while 3n M StIIIβ clustered on a different branch, suggesting that their transcriptome is significantly different from 2n M stIII and 3n M_StIIIα. In F.2, 3n F StIIIα were grouped with female 2n StII and StIII, while 3n F StIIIβ clustered in sub-branch F.1, closer to St0. Overall, these results confirm those of PCA showing that 3nα M oysters are more mature than their corresponding 3nα_F oysters.

Five 3nα StI individuals were branched with males on sub-branch M.1 and the remaining three 3nα StI individuals clustered with females within sub-branch F.1. These latter individuals that clustered with females expressed higher levels of a subset of female-specific genes, including *forkhead box l2* (*foxl2*) and *parathyroid hormone receptor* (*pthr*) (table 1). However, we could not differentiate male and female 3nβ StI. The heat map revealed that 3nβ M StIII in M.2 also expressed high levels of this subset of female-specific genes. Despite this expression of female-specific genes, male-specific genes were also highly expressed in 3n M StIIIβ as in male 2n StIII and 3n StIIIα gonads.

**Table 1:** Subset of female-specific genes that are expressed in 3nβ M.

GenBank Acc	Query length	Best Hit	Species	E value	query coverage
FP010320	387	Similar to parathyroid hormonereceptor 1	*Ciona intestinalis*	8.00E-05	87%
AM860211	680	Forkhead box L2	*Crassostrea gigas*	2.00E-102	98%

### Gene expression along the successive stages of gametogenesis

**Figure 3:**
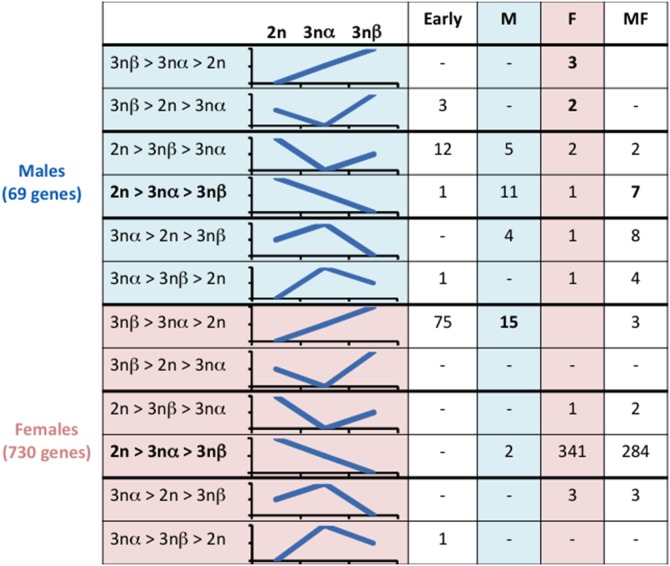
Number of genes differentially expressed within 2n_, 3nα_ and 3nβ_StIII gonads in male (M) and female (F), in both sex (MF) and in early stage (Early) (T test, p<0.05) and their schematical expression profiles.

Studying gametogenesis in diploid oysters previoulsy identified 2,482 genes that were differentially expressed over the course of gonadal development [26] (File S4). To identify factors that contribute towards the differences between 2n, 3nα and 3nβ gonads, we performed a two-way ANOVA on all 2n and 3n StIII, and on male and female individuals (p<0.01, Bonferroni adjusted). We noted a significantly different expression of 69 genes among male 2n, 3nα and 3nβ StIII and of 730 among female 2n, 3nα and 3nβ StIII (File S5, Figure 3). The vast majority of the differentially expressed genes showed a gradual expression pattern from 2n to 3nα to 3nβ with a tendency to be upregulated in 2n and downregulated in 3nβ (20 in males and 625 in females; Figure 3). Genes significantly more expressed in 2n than 3n showed an increased expression over the course of gametogenesis of diploid oysters. The much higher number of genes observed as differentially expressed among females than males is congruent with the greater delay in gametogenesis of 3n among females than in males shown by PCA.

Respectively in females and males, 93 and 8 of the genes differentially expressed were significantly upregulated in 3nβ by comparison with 2n and 3nα and more specifically, 75 and 3 of them in early gametogenetic stages (cluster 1). Among those, we found genes involved in the control of cell growth and cell division such as a *serine protease htra1* and *glypican-5*. In female 3nβ, we also found upregulated the *bone morphogenetic protein 2* (*bmp2*), a member of the TGF-β superfamily involved in ovarian development and in male 3nβ, a *beta catenin* that contributes towards stopping cell division. However, we also found 15 male-specific genes upregulated in female 3nβ including genes involved in spermatogenesis such as *enkurin*, *slit homolog 2* and *elav-like*. Conversely, we also found 5 female-specific genes, including *forkhead box l2* (*foxl2*), upregulated in male 3nβ (table 1; Figure 3).

Surprisingly, some genes were upregulated in 3nα compared to other types of oysters (Figure 3). In male 3nα, we identified 19 upregulated genes among which 4 were male-specific (*slit homolog 2 protein*, *serine palmitoyl transferase*, *ring finger protein* and *ef-hand calcium-binding protein*). Other upregulated genes included *thyroid receptor-interacting protein 13* (*trip13*) and *f box only protein 43* required for meiosis, and *cep57-related protein* involved in microtubule attachment to the centrosome. Female 3nα displayed upregulated expression of only 7 genes including *transforming growth factor beta induced protein ig-h3* involved in the control of cell proliferation and differentiation, the maternal *dna replication licensing factor mcm3* (Mini-Chromosome Maintenance protein 3) that allows DNA to undergo a single round of replication per cell cycle, and *smarca1* (*swi/snf related*, *matrix associated*, *actin dependent regulator of chromatin*, *subfamily A*, *member 1*) that encodes the probable global transcription activator SNF2L1 (Sucrose Non-Fermenting 2-like protein1).

## Discussion

Polyploidy generally has a strong effect on the physiology of animals [1], [2], [3] and often renders them sterile or able to reproduce by parthenogenesis only [8], [9], [10]. Among the polyploids, triploids are less commonly observed in the wild because of the odd number of chromosomes, that severely impacts mitosis and meiosis [32], [33]. As a result, triploidy is often associated with asexual reproduction, such as in loaches, frogs and lizards.

To our knowledge, this is the first comprehensive study of gene expression during gametogenesis of triploid in any organism. Pacific oysters are of particular interest in this respect, because triploid oysters show diverse abilities to produce gametes [20]. We were particularly interested in investigating the unusual ability of 3nα individuals to perform advanced gametogenesis by comparing them to 3nβ individuals, which show less developed gonadic tubules [20], and to 2n individuals displaying normal gonadal development [26].

To this end, we used a genome wide microarray design and previously developed and validated protocols [25]. Using applied principal component analysis, we then discriminated individuals depending on stages of mitosis and gonadal development, ploidy and sex. After analyzing the effect of triploidy on the gonad tissues, we then evaluated the differences observed in the transcriptome of 3nα and 3nβ male and female gonads compared to 2n gonads with a view to adressing the following fundamental questions:

### (1) Are there genes differentially expressed in gonads of 2n and 3n regardless of their developmental stage and sex?

Physiological differences between 2n and 3n oyster gonads can only be observed from stage I for 3nβ and from stage II for 3nα [20]. However, the direct comparison of 2n and 3n gonad transcriptomes identified 1,911 genes differentially expressed from St0 to StIII gonads. Interestingly, more genes were downregulated than upregulated in 3n.

Upregulated genes in gonads of triploid oysters organized themselves around the NF-kappaB complex. NF-kappaB has been implicated, *inter alia*, in the DNA damage response [34], the activation of which controls the transcription of cell survival genes, allowing cells to escape apoptosis and to initiate DNA repair. Indeed, NF-kappaB induces the expression of cellular inhibitors of apoptosis (c-IAPs) that bind and inhibit caspase-3 and -7 [35]. The observed upregulation of both *caspase-3* and -*7* as well as *hint1* in 3n, would suggest an important role for apoptosis, as discussed by Jouaux *et al*. [20]. However, the concurrent upregulation of inhibitors of apoptosis *c-iap2/birc3* (*baculoviral iap repeat containing protein 3*) and *anamorsin* (named also *cytokine induced apoptosis inhibitor 1* or *ciapin1* [36]) in 3n rather indicates the presence of a balanced system regulating DNA repair/apoptosis in these cells.

Downregulated genes in triploid oysters were in linked in some way to *rock* (Rho-associated protein kinase) encoding the actin cytoskeleton protein ROCK, and *smarca4* (SWI/SNF related, matrix-associated actin-dependent regulator of chromatin, subfamily A, member 4). Numerous other actin and actomyosin assembly proteins were also downregulated in the gonad. The downregulation of *rock* and *smarca4* genes would be expected to affect numerous steps of gametogenesis including cell polarity, spindle formation, chromosome segregation, cytokinesis, polar body exclusion and gamete maturation [37], [38], [39], ultimately leading to G1 arrest [40]. Germ cell development also relies on chromatin remodeling and a downregulation of genes such as *smarca4* can result in arrest during prophase of meiosis [41]. Others genes involved in the SWI/SNF chromatin remodeling complex were also downregulated in 3n, such as those encoding AT-rich interactive domain-containing proteins, *arid1* and *arid2*, and the SWI/SNF complex subunit, *smarcc2*. All together these results suggest a significant reduction of cell division in gonads of triploid oysters via epigenetic mechanisms. This last hypothesis is supported by the distinct profiles of DNA condensation between 2n and 3n observed during their gametogenesis [20].

Overall, our comparative analysis has revealed an upregulation in 3n oyster gonads of genes mostly involved in DNA repair and apoptosis, in accordance with the previous observation of apoptosis events in triploid oyster gonadal tubules [20], and a downregulation of those mostly involved in cell division and its transcriptional and posttranscriptional regulation.

### (2) What delays gametogenesis in 3nβ?

In order to characterize the factors that hinder gonad development in 3nβ individuals, we specifically searched for genes upregulated in 3nβ individuals. Among those found, 75 and 3 genes were also upregulated in early gametogenetic cycles in 2n females and males respectively, suggesting a simple delay in gametogenesis in the 3nβ individuals. In females, upregulated genes included numerous genes able to limit cell growth including *glypican-5*, *htra1*, *bmp2*. In mammals, Glypicans regulate the several signaling pathways that play critical roles in morphogenesis [42]. In Drosophila, the glypican *dally* is expressed in and maintains the female germline stem cell niche [43]. The serine protease HTRA1 regulates the availability of insulin-like growth factors and regulates cell growth. More specifically it inhibits the signaling of *bmp2* [44] which encodes the bone morphogenetic protein 2, a member of the TGF-β superfamily expressed specifically in XX gonads in mammals and involved in ovarian development [45]. Such upregulation of growth inhibitors may cease the division of cells, and would explain why little or no proliferation occurs in 3nβ [20].

Surprisingly, some genes upregulated in 3nβ_StIII_F were previously found to be male-specific, and conversely other genes upregulated in 3nβ_StIII_M are female-specific genes [26]. 3nβ_StIII_F showed an upregulation of 15 genes previously associated with spermatogenesis in *C. gigas* such as *enkurin*, *slit homolog 2* and *elav-like* [26]. Enkurin is an adaptor that interacts with transient receptor potential cation channels and mediates Ca2+ entry into the cells, a process necessary for flagellar motility in spermatozoids. In vertebrates, *slit homolog 2* and *elav-like* (ELAV, embryonic lethal, abnormal vision) are involved in neuronal development and differentiation [46], [47], however, their male-specific expression in oysters suggests a role in spermatogenesis [26]. Interestingly, in the flatworm *Macrostomum lignano*, an *elav-like* gene is necessary for successful spermatid differentiation [48]. Male 3nβ displayed upregulated expression of at least four female-specific genes, including *foxl2* from StI to StIII. *Forkhead box l2* is the earliest known sex dimorphic marker of ovarian determination/differentiation in vertebrates [49] and prevents the differentiation of ovaries to testes in mammals [48]. It was found to be female-specific in *C. gigas* [26], [50]. Most interestingly, the natural antisense transcript of *foxl2* seems to be involved in the regulation of oyster sex determinism [51]. Other genes upregulated in 3nβ_StIII_M included *β-catenin*, involved in female sex determination and in mammals encoding the key pro-ovarian and anti-testis signaling molecule β-Catenin [52].

In diploid oysters, both male and female germ cells proliferate mitotically during StI of gonadal development until a “transition step” when they cease to divide before initiating meiosis. In 2n individuals, physiological differences between male and female can be observed from this stage and allow sex determination by histological methods [27], [28]. Interestingly, physiological differences between 2n and 3n gonads can be observed from StI despite the sex of these individuals remaining difficult to determine. Our results suggest that while it is possible to determine the sex of 3nα_StI, distinguishing male and female 3nβ_StI remains problematic. Regulators controling growth and the mitosis/meiosis decision are often the same as those controlling sex determination. In 3nβ individuals, the disruption of sex differentiation may explain the blocking of gametogenesis at this early stage.

### (3) How do 3nα succeed in producing mature and viable gametes despite the presence of a third set of chromosomes?

Disruption of gonadal development may occur in triploids due to the presence of a third set of chromosomes interfering with the normal pairing of homologous chromosomes in meiotic prophase, which inhibits further gamete development. Guo and Allen [53] analyzed the number of chromosomes in triploid gametes and observed a distribution of aneuploidy that can be explained by a random segregation of the extra chromosome during anaphase I. In the present study, most genes that were upregulated in 3nα were also downregulated in 3nβ by comparison with 2n. This apparent over compensation may allow 3nα to overcome the blocking of gametogenesis and to deal with the third set of chromosomes. Indeed, we detected an upregulation of the genes encoding the maternal DNA replication licensing factor MCM3 and the probable global transcription activator SNF2L1 in 3nα_StIII_F. MCM3 is a replicative helicase involved in cell cycle check point and essential for ‘once per cell cycle’ DNA replication during mitosis [54]. The probable global transcription activator SNF2L1, encoded by the gene *smarca1*, is a helicase thought to regulate transcription of genes by altering the chromatin structure around these genes, suggesting an epigenetic reprogramming to handle triploidy. Expressed in human oocytes [55], inhibition of SNF2L1 leads to DNA damage and growth inhibition [56]. We also observed an upregulation of *ig-h3* that is induced by the transforming growth factor beta (TGF-β), the signaling of which is essential for oogenesis [57]. Also, in 3nα_StIII_M, we observed an upregulation of the genes encoding TRIP13 and Cep57-related proteins. TRIP13 is an essential component of the synapsis checkpoint that is required for completion of meiosis in both sexes from yeast and mouse [58], [59]. This pachytene cell cycle checkpoint monitors the fidelity of homologous chromosome synapsis, segregation and recombination. Cep57 is a centrosomal protein required for microtubule attachment to centrosome [60]. In its absence, bipolar spindles appear elongated and the cell fails to align chromosomes, a necessary step before segregation during cell division [61]. Overall, in 3nα individuals, we observed an upregulation of genes that can be attributed to the regulation of DNA replication, recombination and pachytene checkpoint, that is likely responsible not only for the delay in gametogenesis displayed by 3nα with respect to 2n, but also for their success in producing some viable gametes despite the presence of a third set of chromosomes [62].

## Conclusions

This comparative transcriptomic analysis has shown an increase in DNA repair and apoptosis in triploid gonads and a decrease in cell division, indicating a DNA checkpoint control over cell integrity. Gametogenesis in 3nβ individuals would appear to be slowed down due to an impaired sex differentiation brought about by the presence of the third set of chromosomes. However, 3nα individuals succeed in producing gametes, at least in part, by virtue of the pachytene checkpoint that, by preventing nuclear division until the cell succeeds in chromosome synapsis, prevents missegregation of chromosomes resulting in a random segregation of the extra chromosome [53].

Despite all this new information, the mechanisms regulating gametogenesis in triploid individuals are far from being fully elucidated. It still remains to be determined whether the 3nα or 3nβ status is determined genetically. Our results suggest that this question is closely related to that surrounding sex determination in oyster being controlled genetically or environmentally. Analyses of *C. gigas* sex ratio suggests that sex is mostly determined environmentally and mediated via temperature and individual energy allocation [18], [63]. In line with this hypothesis, we could argue that 3n oyster success in gametogenesis be also under the control of temperature and food availability [27], [63], [64]. It will be interesting to address this question by monitoring the sex and reproductive investment of a single group of triploid oysters, over successive years and under different environmental constraints.

## Supporting Information

File S1 **Technical validation of expression profiles by real-time quantitative PCR.** The table provides the list of genes used for qPCR validation, and their accession number, description and primers sequences. The figure illustrates the gene expression by RT-qPCR, in comparison to the expression values obtained by the microarray analysis.
(DOCX)

File S2 **List of genes differentially expressed between diploids and triploids.** This table provides the list of genes differentially expressed between diploid and triploid oysters (Student’s t-test with Bonferroni adjustment. ID_Ref: Identity of the spot on the microarray; Genbank: Genbank accession number; Description: description as uploaded from Sigenae (http://www.sigenae.org); 2N Mean: mean of log normalized expression values of diploids; 2n StDev: standard deviation of log normalized expression values of diploids; 3N Mean: mean of log normalized expression values of triploids; 3N StDev: Standard deviation of log normalized expression values of triploids; Abs t value: Absolute t value; df: degrees of freedom; Raw p value; Adj p value: Adjusted p value; clusters: Clusters obtained by K-means clustering; second last column: difference of expression between 2N and 3N; last column: categories of ploidy where gene is overexpressed.
(XLS)

File S3 **Genes overexpressed and underexpressed in triploid oyster gonads compared to diploid oyster gonads.** Functional classification with Ingenuity Pathway Analysis (IPA) with the lists of upregulated and downregulated genes.
(DOCX)

File S4 **List of genes that were differentially expressed over the course of gonadal development.** This file provides the list of genes differentially expressed between diploid and triploid gametogenetic stages (two-way ANOVA on all 2n and 3n StIII, and on male and female individuals (p<0.01, Bonferroni adjusted). spot: Identity of the spot on the microarray; ID: Genbank accession number; Description: description as uploaded from Sigenae (http://www.sigenae.org); gameto: stage of gametogenesis; tissus: tissu specific of the gene; ST0, ST1, ST2 and ST3: diploid gametogenetic stages followed by the sex (F: female and M: male) and the number attributes at each individual; 3N O, 3N 1 and 3N 3: triploid gametogenetic stages followed by the blockade status ( α: alpha and β: beta) and sex (F: female and M: male) and the number attributes at each individual).
(XLSX)

File S5 **List of different genes between 2n, 3nα and 3nβ gonads.** This file provides the list of genes that contributes towards the differences between 2n, 3nα and 3nβ gonads (Two-way ANOVA on all 2n and 3n StIII, and on male and female individuals (p<0.01, Bonferroni adjusted)). spot: Identity of the spot on the microarray; ID: Genbank accession number; Description: description as uploaded from Sigenae (http://www.sigenae.org); gameto: stage of gametogenesis; tissus: tissu specific of the gene; 2N Mean: mean of log normalized expression values of diploids; 2n StDev: standard deviation of log normalized expression values of diploids; 3Na Mean: mean of log normalized expression values of alpha triploids; 3Na StDev: Standard deviation of log normalized expression values of alpha triploids; 3Nb Mean: mean of log normalized expression values of beta triploids; 3Nb StDev: Standard deviation of log normalized expression values of beta triploids; F ratio; SS (groups): Sum of square between groups; SS (error): Sum of squares of error; df (groups): degrees of freedom for groups; df (error): degrees of freedom for error; raw p value; Adj p value: Adjusted p value.
(XLS)
