# Supplementary material for: Transcriptomic Profiling of Gametogenesis in Triploid Pacific Oysters Crassostrea gigas: Towards an Understanding of Partial Sterility Associated with Triploidy
Source: PLoS One. 2014 Nov 6;9(11):e112094. doi: 10.1371/journal.pone.0112094 (PMC4222980; doi:10.1371/journal.pone.0112094)
Supplement: File S1 — Technical validation of expression profiles by real-time quantitative PCR. The table provides the list of genes used for qPCR validation, and their accession number, description and primers sequences. The figure illustrates the gene expression by RT-qPCR, in comparison to the expression values obtained by the microarray analysis. (DOCX) [file pone.0112094.s001.docx]

**Supplementary File 1 :** Technical validation of expression profiles by real-time quantitative PCR

Table: Primer sequences used for real-time quantitative PCR

| Accession number | Description | Forward / reverse primers |
| --- | --- | --- |
| AM860211 | Forkhead box protein L2 | CTCAGGATCTGTATATTTGT  GAAGAAGAATTTGTAGAGAC |
| AM868677 | Catenin beta | CCATGATGGTTCACCAGTTG  ACAGCGAGTGGTCTCCAAGT |
| ES789247 | Enkurin | AATTGATCAGGTAAAATTGCTCATAA  GAACCTCAGAAGTGGAAAACAATT |
| CU991158 | unannotated | TCGTTCTGTATTCTGTGGAGTTG  GGGTTTTGAACAATTTTCCATC |
| AM860913 | DSX | CAGTCCTCCACACACCTGTC  AAGGATGGGGTGGAGAAAAC |
| AM867021 | ELAV-4 | GCCGTTCAGAATGTCAAGGT  ACGGAATGTTGTTGTTGAAATG |
| DV736924 | unannotated | CCGTACATTCTCGCCTCATT  ACGGAATGTTGTTGTTGAAATG |
| AM858268 | unannotated | TTGCCTTACTGGGAAATTACG  TCTGTTCTTGTGCTGGTTCG |


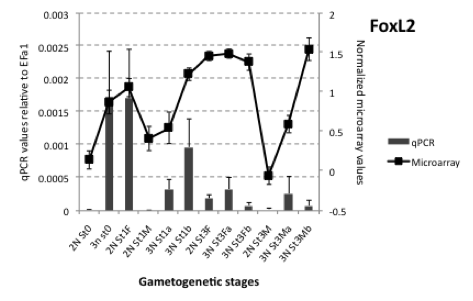

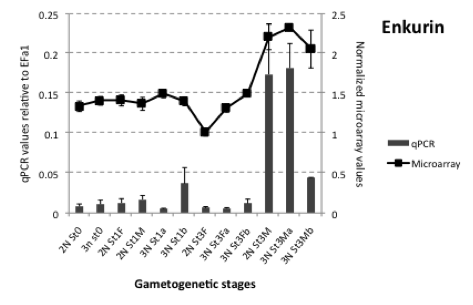

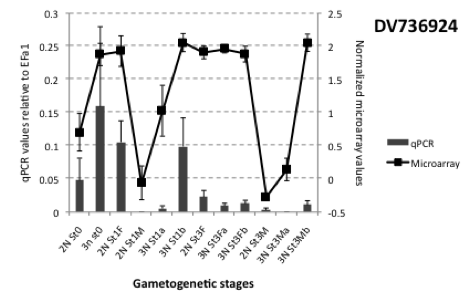

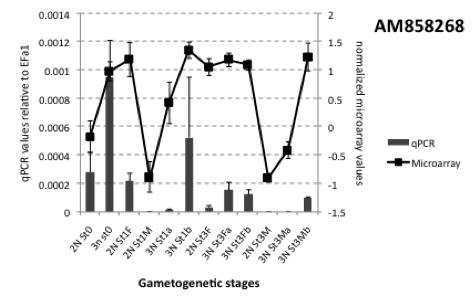

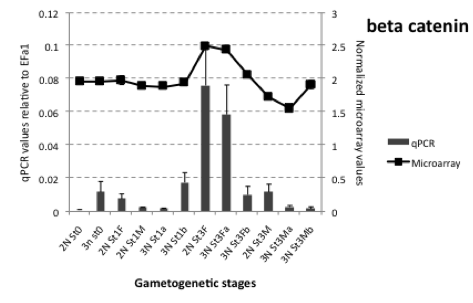

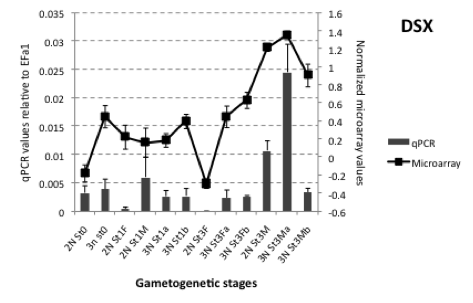

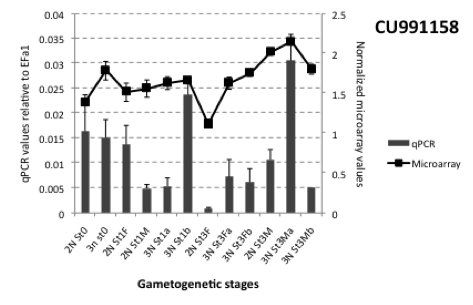

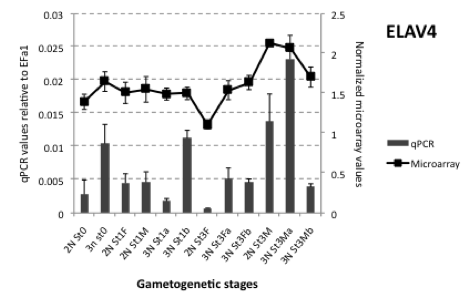


Figure: Microarray and quantitative PCR expression profile of selected genes. The real time qPCR values are displayed on left axis (histograms) and normalized to efa1 gene expression and the microarray log2 transformed values are indicated on right. Bar on histograms and curves represent standard deviation. On horizontal axis, are displayed the gametogenetic stages from 2n and 3n animals. (St 0: resting period; StI: mitosis proliferation; stIII: maturation; a: 3n α and b: 3n β animals).
