# Supplementary material for: Transcriptomic Profiling of Gametogenesis in Triploid Pacific Oysters Crassostrea gigas: Towards an Understanding of Partial Sterility Associated with Triploidy
Source: PLoS One. 2014 Nov 6;9(11):e112094. doi: 10.1371/journal.pone.0112094 (PMC4222980; doi:10.1371/journal.pone.0112094)
Supplement: File S3 — Genes overexpressed and underexpressed in triploid oyster gonads compared to diploid oyster gonads. Functional classification with Ingenuity Pathway Analysis (IPA) with the lists of upregulated and downregulated genes. (DOCX) [file pone.0112094.s003.docx]

Supplementary File 3: Genes overexpressed and underexpressed in triploid oyster gonads compared to diploid oyster gonads.

Genes involved in cell cycle, protein synthesis and protein trafficking tend to be more expressed in 3n (table 1). In contrast, genes involved in cell morphology, molecular transport, lipid metabolism, cellular compromise and movement, cell and tissue development tend to be less expressed in triploid gonads than in diploid ones (table 1).

Table 1: Functional classification of genes differentially expressed between triploid and diploid oyster gonads. The table provides the total number of proteins affected, the total number of genes upregulated and downregulated in triploids, the number of genes with a predicted function assigned, and the proportion (%) of the annotated genes within each functional category as predicted using Ingenuity Pathway Analysis.

| **Functional classification** | **proteins affected** | **upregulated** | **downregulated** |
| --- | --- | --- | --- |
| **Total** | **1,911** | **505** | **1,406** |
| **annotated** | **730** | **260** | **470** |
| Cell death | **11.6** | 9.6 | **12.8** |
| Cellular assembly and organization | **9.0** | 4.2 | **11.7** |
| Small molecule biochemistry | **6.0** | 3.5 | **7.4** |
| Cell morphology | 4.9 | 3.5 | **5.7** |
| molecular transport | 4.9 | 3.8 | **5.5** |
| Cellular development | 4.2 | 2.3 | **5.3** |
| Lipid metabolism | 3.8 | 2.3 | **4.7** |
| Cell cycle | 3.7 | **3.8** | 3.6 |
| Cellular compromise | 3.4 | 2.7 | **3.8** |
| Cell movement | 3.4 | 1.9 | **4.3** |
| Tissue development | 3.3 | 1.2 | **4.5** |
| Cellular function and maintenance | 2.9 | 1.9 | **3.4** |
| Protein synthesis | 2.7 | **3.5** | 2.3 |
| Protein trafficking | 2.6 | **3.1** | 2.3 |
| RNA post-transcriptional molecules | 2.6 | **2.7** | 2.6 |
| Connective tissue development and function | 2.6 | 0.8 | **3.6** |
| Cell to cell signaling and interaction | 2.5 | 1.9 | **2.8** |
| Carbohydrate metabolism | 2.2 | 0.0 | **3.4** |
| Cell growth and proliferation | 2.1 | 1.5 | **2.3** |
| Embryonic development | 2.1 | 0.4 | **3.0** |
| Post translational modification | 2.1 | 1.5 | **2.3** |
| Cell signaling | 1.8 | 1.2 | **2.1** |
| Nucleic acid metabolism | 1.5 | **1.5** | 1.5 |
| Protein degradation | 1.5 | **2.3** | 1.1 |
| DNA replication, recombination and repair | 1.5 | **2.3** | 1.1 |
| Gene expression | 1.4 | 0.8 | **1.7** |
| Tissue morphology | 1.2 | 0.4 | **1.7** |
| amino acid metabolism | 1.2 | 0.4 | **1.7** |
| Organ development | 1.0 | 0.0 | **1.5** |
| Tumor morphology | 0.7 | **1.2** | 0.4 |

The three networks more relevant to the original list of focus genes are represented as graphs indicating the molecular relationships between genes. Genes are represented as nodes and the biological relationships between genes are represented as lines or arrows. The intensity of the node color indicates the degree of up (red) or down (green) regulation. Genes in uncolored nodes were not identified as differentially expressed in our experiment but were algorithmically integrated into the network on the basis of evidence stored in the IPA knowledge memory.


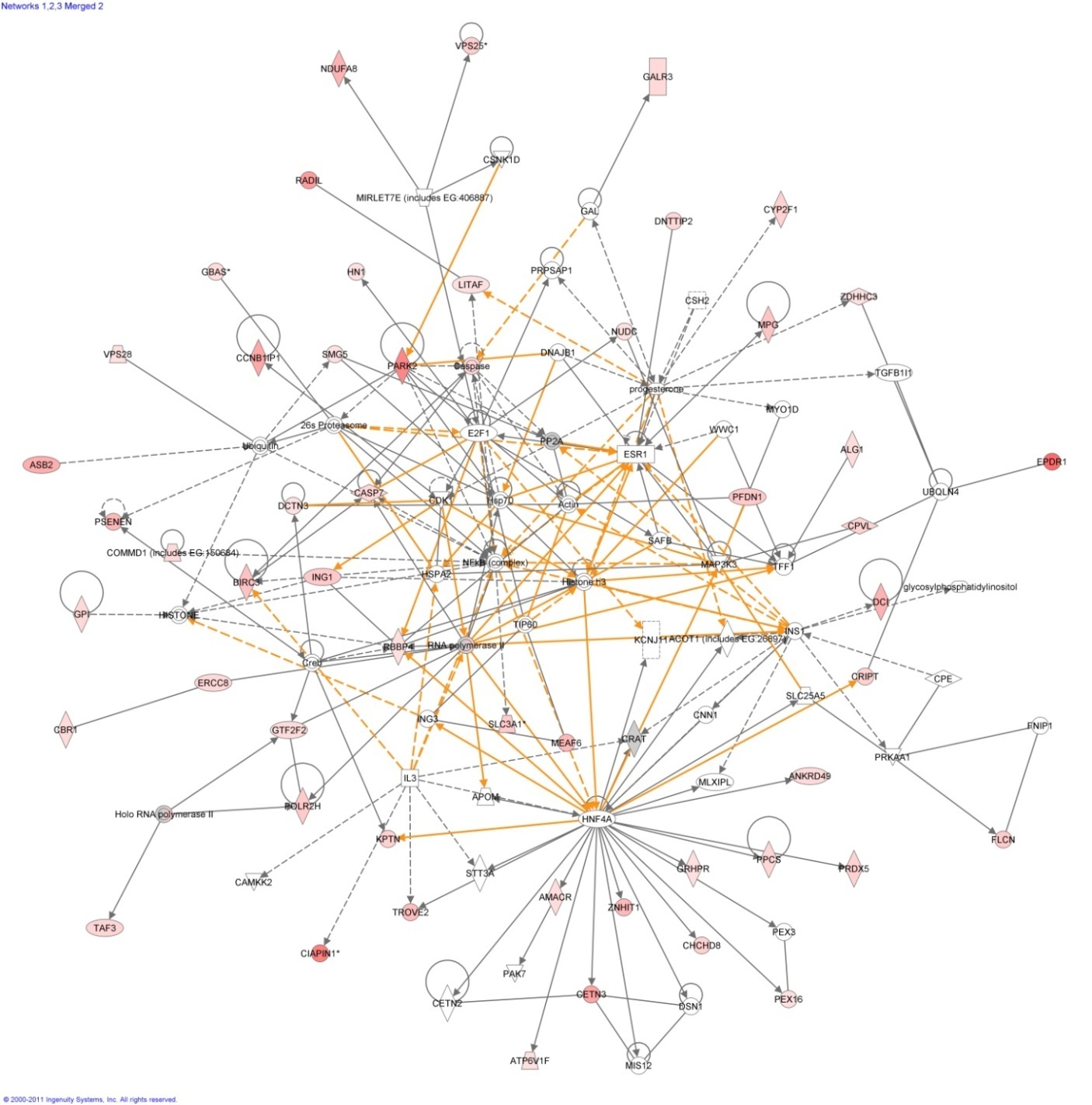


Figure 1: Merged network of the most significant 3 gene networks of genes overexpressed in triploid oyster gonads compared to diploid oyster gonads. The intensity of the node color indicates the degree of upregulation. Nodes are displayed using various shapes that represent the functional class of the gene product.: enzymes (◊), phosphatases (🛆), kinases (∇), peptidases (), G-protein coupled receptor (🗌), transmembrane receptor (), cytokines (□), growth factor (□), ion channel (🗌), transporter (), translation factor ( ), nuclear receptor (), transcription factor () and other (🌕).


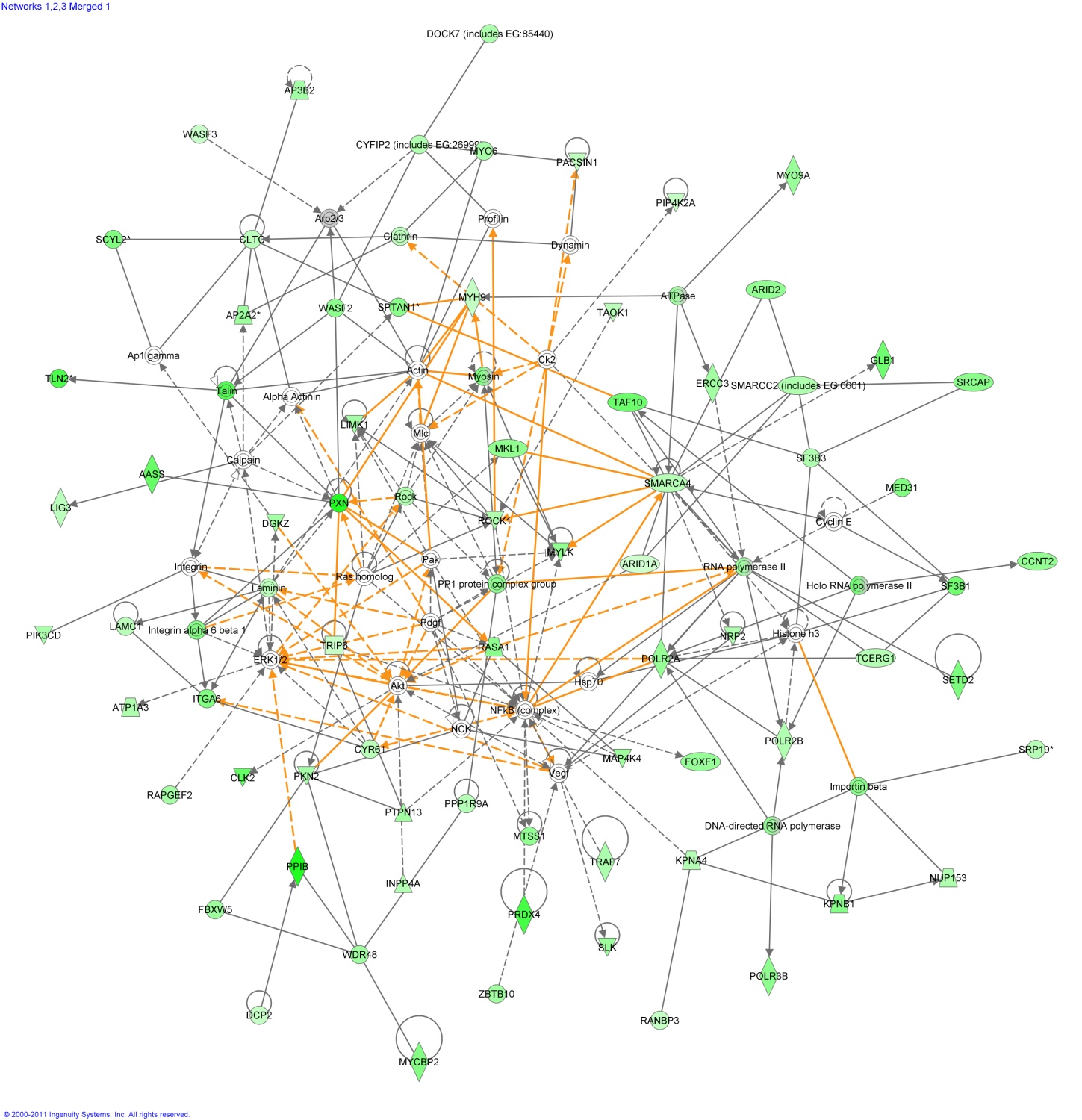


Figure 2: Merged network of the most significant 3 gene networks of genes underexpressed in triploid oyster gonads compared to diploid oyster gonads. The intensity of the node color indicates the degree of upregulation. Nodes are displayed using various shapes that represent the functional class of the gene product.: enzymes (◊), phosphatases (🛆), kinases (∇), peptidases (), G-protein coupled receptor (🗌), transmembrane receptor (), cytokines (□), growth factor (□), ion channel (🗌), transporter (), translation factor ( ), nuclear receptor (), transcription factor () and other (🌕).
